# Efficacy of treatment with glucosamine sulfate in patients with knee effusion due to osteoarthritis

**Published:** 2013

**Authors:** Murat Korkmaz, Fatih Karaaslan, Yalcin Erdogan, Esef Bolat, Seyhan Karacavus, Hafize Kizilkaya, Ilhan Gunaydin

**Affiliations:** 1 Murat Korkmaz, Assistant Professor, Department of Orthopaedics and Traumatology, Bozok University Medical Faculty, Yozgat, Turkey.; 2 Fatih Karaaslan, Assistant Professor, Department of Orthopaedics and Traumatology, Bozok University Medical Faculty, Yozgat, Turkey.; 3 Yalcin Erdogan, Assistant Professor, Department of Family Medicine, Bozok University Medical Faculty, Yozgat, Turkey.; 4 Esef Bolat, Assistant Professor, Department of Anesthesiology, Bozok University Medical Faculty, Yozgat, Turkey.; 5 Seyhan Karacavus, Assistant Professor, Department of Nuclear Medicine, Bozok University Medical Faculty, Yozgat, Turkey.; 6 Hafize Kizilkaya, Medical doctor, Department of Internal Medicine, Bozok University Medical Faculty, Yozgat, Turkey.; 7 Ilhan Gunaydin, Professor, Department of Internal Medicine - Rheumatology, Bozok University Medical Faculty, Yozgat, Turkey.

**Keywords:** Anti-inflammatory, Effusion, Glucosamine Sulfate, Osteoarthritis

## Abstract

***Objective: ***Evaluation of anti-inflammatory effect of Glucosamine sulfate (GS) versus diclofenac sodium (DS) in effusion of osteoarthritic knees.

***Methodology: ***In this study, patients were included in this study from 2007-2010 based on American College of Rheumatology criteria with OA and physical examination in effusion of osteoarthritic knees. The patients were divided into two groups. First group (27 patients) DS was given in doses 75 mg twice daily for ten day. In the group II (25 patients) GS was used in doses of 1500 mg two times daily over the first 12 weeks of the study. A closed aspiration was performed. The knee circumference was measured in patients before and 12 week after treatment. Before and after 12 weeks of treatments, both groups of patients were assessed according to the WOMAC questionnaire of knee pain and function scores.

***Results: ***Comparison of knee mean circumference between the two groups was not statistically significant before treatment (p=0.938), but significant after treatment (p<0.001). At the end of the 12 week, there was 66.6% complete resolution of effusion in the DS group (18 patients) and 24.0% (6 patients) in the GS group, this was statistically significant (*P<*0.001). DS groups, results of the beginning and at the end of 12 week measurement showed significant differences in WOMAC pain mean score (P < 0.001) but GS groups not statistically significant (P=0.160). The WOMAC function mean scores in pre and post-treatment periods of follow-up showed significant variation between the two groups (P< 0.001, P<0.001).

***Conclusions:*** Our observations suggest that GS is not able to suppress the progression of adjuvant arthritis in OA with effusion of knee osteoarthritis. GS should not be expected as anti-inflammatory influence as DF in the treatment of OA-related effusion.

## INTRODUCTION

Arthritis continues to be a large public health problem.^1^ Osteoarthritis (OA) is the most common type of arthritis. More than 27 million adults in the United States have OA, a number likely to increase due to the obesity, epidemics and aging of the population.^2^ The disease affects the cartilage, synovium, subchondral bone, tendons and muscles surrounding the joint. As clinical symptoms, pain and limited range of motion is frequently associated with joint effusion.^3^ Effusions in knee with OA is often treated with non-steroidal anti-inflammatory drugs (NSAID).^3^^,^^4^ Among the NSAID‘s diclofenac sodium (DS), is frequently usesd in the treatment of these patients.

Many people are trying new therapies and dietary supplements such as glucosamine and chondroitin sulfate for treatment of OA. Glucosamine is an aminosaccharide, acting as a preferred substrate for the biosynthesis of glycosaminoglycan chains and subsequently, for the production of aggrecan and other proteoglycans of cartilage.^5^ Glucosamine sulfate (GS) reduced knee pain and improved muscle strength with resistance training but their effects on cartilage and synovium metabolism in patients with OA are controversial.^6^ Loss of minimum joint space width over two years was significantly lower in Glucosamine sulfate (GS) group than placebo graph. However, there was no significant evidence in favour of trials with GS having positive outcomes in effusion of knees OA.^7^^,^^8^

The purpose of this study was to compare efficacy of, treatment of effusion of knees caused by OA with GS versus NSAID.

## METHODOLOGY

In this study, patients were included in the study group between January 2007 - December 2010, based on American College of Rheumatology (ACR) criteria with synovitis on physical examination of OA.^9^ Exclusion criterias were: knee trauma during the previous month; inflammatory synovitis (infection or other rheumatic diseases), intraarticular injections (corticosteroids, viscosupplementation) during the previous 3 months.

The patients were divided into two groups. First group (27 patients) DS was given in doses of 75 mg twice daily with breakfast and after supper for ten days. In group II (25 patients) GS was used in doses of Glucosamine sulfate 1500 mg (Dona sase 1500 mg Glucosamine sulfate Rottapharm Ltd. - Irlanda) two times daily over the first 12 weeks of the study. Knee circumferences were measured just above the superior border of patella at the beginning and at the end of a month. The knee circumference was measured in patients before and after 12 week treatment. According to Kellgren-Lawrence classification radiographs were graded for OA changes in all patients.^10^ At beginning of treatment, a closed aspiration was performed in all patients for discharge with knee effusion. Patients were evaluated both in the beginning and at the end of study period using Western Ontario and McMaster Universities Osteoarthritis Index (WOMAC) questionnaire of knee pain and function scores.^11^

SPSS 15.0 was used for statistical analysis and the variables were compared by using chi-square and Friedman tests. Values less than 0.05 were considered significant.

## RESULTS

The mean age was 56.6±1.1 years in group I and 57.2±0.8 years in group II. The detailed demographic and baseline clinical characteristics between the two groups is shown in Table-I. There were no significant differences in pre treatment characteristics operative factors between the two groups.

**Table-I:** Demographic and baseline clinical characteristics of patients

	*Diclofenac sodium * *(n=27)*	*Glucosamine sulfate (n=25)*
Men/Women n(%)	8(29.6)/19 (70.4)	8(25.0)/17(75.0)
Age, years ±SD	56.6±1.1	57.2±0.8
Body mass in kg/m2±SD	27.5 ±2.0	27.5 ± 2.1
Duration of synovitis day ±SD	21.4±0.2	16.7±03
Kellgren-Lawrence grade n(%)		
2	14 (51.9)	12 (48.0)
3	8 (29.6)	7 (28.0)
4	5 (18.5)	6 (24.0)

n: number SD: standard deviation kg/m2:Kilogram/square meter

**Table-II:** Synovial effusion measurement and change of treatments

	*Diclofenac sodium (n=27)*	*Glucosamine sulfate (n=25)*	*P Value*
Knee circumference (cm±SD)			
Before treatment	45.9 ±2.8	45.7± 1.2	=0.923
After treatment	41.2±2.5	44.2±2.2	<0.001
Complete resolution of SE n(%)	6(11.3)	18(35.2)	<0.001
SE amount of discharged (ml)	22.8	25.7	=0.876

cm: centimeter SD: standard deviation n: number SE: Synovial effusion ml:milliliter

**Table-III:** Knee circumference measurement and change of treatments

*Knee circumference*	*Before treatment (cm) ± SD*	*After treatment (mm) ± SD*	*p value*
Diclofenac sodium (n=27)	45.9 ±2.8	41.2±2.5	<0.001
Glucosamine sulfate (n=25)	45.7± 1.2	44.2±2.2	=0.938

cm: centrimeter SD: standard deviation n: number

**Table-IV:** WOMAC mean change from baseline in the primary and secondary outcome measure

*WOMAC*	*Diclofenac sodium * *(n=27)*	*Glucosamine sulfate (n=25)*	*p value*
*Pain*			
Before treatment	19.55±2.9	19.40±2.3	=0.831
After treatment	12.70±5.1	18.44±2.7	<0.001
P value	<0.001	= 0.160	
*Function*			
Before treatment	50.51±7.3	51.56±8.5	= 932
After treatment	27.66±7.5	33.12±6.4	= 206
P value	<0.001	<0.001	

n: number

In terms of quantity of joint effusion, the quantity of joint synovial fluid was an average of 22. 8 ml in the group I and an average of 25.7 ml in the group II when punctured before the drug treatment. Overall range: 5-70 ml of synovial fluid was present in the joints. There was no significant difference in quantity of joint effusions between two groups before administration (p=0.748).

Comparison of knee mean circumference between the two groups was not statistically significant before treatment (p=0.938), but significant after treatment p<0.001). At the end of the 12 week, there was 66.6% complete resolution of knee effusion in the DS group (18 patients) and 24.0% (6 patients) in the GS group, this was statistically significant (P<0.001). (Table II and III)

DS groups, results at the beginning and at the end of 12 week measurement showed significant differences in WOMAC pain mean score (P < 0.001) but in the GS groups were not statistically significant(P=0.160). The WOMAC function mean scores in after and before periods of follow-up showed significant variation between the two groups (P< 0.001, P<0.001) (Table-IV)

As a side effect which seemed to be caused by administration of group I, one case of “tip II diabetes mellitus” was reported. The symptoms were beginning and improved immediately after discontinuation of administration. One of the patients (4%) in group II and four patients (14.2%) in group I experienced an adverse gastrointestinal (GI) symptoms (P<0.001). This showed that GI symptoms were more in the DS group.

## DISCUSSION

Although GS treatment has been used for years with OA, there are conflicting results of this treatment in effusion with OA. In this study we administered GS to OA patients and evaluated its effect on effusion which is used to compare the anti-inflammatory properties of DF. These were assumed to have improved as a result of improvement in joint effusion by GS but this effect is not as good as DF.

Intra-articular corticosteroid application is one of the method used for treatment of effusion with knee OA. That is recommended in several guidelines for treatment of patients with knee synovit and effusion due to OA.^12^^,^^13^ It showed abnormal stress responses due to hypothalamus pituitary-adrenal suppression, accelerated joint destruction, septic arthritis and needed repeated injections which limit the usefulness of these agents for management of knee OA with effusion.^14^^-^^16^

NSAIDs are commonly used in OA with knee effusion. In patients with a flare up of knee OA, specifically in patients with evidence of joint inflammation documented by knee swelling, there was a significant increase in markers reflecting cartilage and synovium metabolism that could partly be prevented by high doses of NSAIDs.^17^ NSAIDs diclofenac sodium is frequently used effectively in the treatment of OA.^18^ The widespread and long term use of NSAIDs among elderly people with OA is associated with considerable side effects. NSAIDs cause serious gastrointestinal complications such as bleeding or perforation.^19^ This drug can reduce pain in short periods only slightly better than placebo but may inhibit long-term healing of damaged cartilage and serious adverse effects.^20^ Our study showed that GI symptoms were more in the DS group patients.

GS is widely used to treat or prevent osteoarthritis in humans.^21^ Several short- and long term clinical trials in osteoarthritis have shown the significant symptom-modifying effect of glucosamine.^22^ Some data showed that glucosamine can prevent experimentally induced cartilage degradation.^23^ Hua et al reported, microscopically and biochemically the effect of glucosamine, suppressed not only the increase in the swelling of joints and the arthritis score but also the histopathological changes of joints in adjuvant arthritis (synovial hyperplasia, pannus formation with cartilage erosion, and leukocyte infiltration.^7^ But, on the contrary as clinically, our study had no effect in the GS group patients.

Our study had some limitations. Placebo group as predefined in the original trial, decreased power to detect significant difference with the active treatment group.

## CONCLUSIONS

Our observations suggest that GS is not able to suppress the progression of adjuvant arthritis in OA with effusion of knee osteoarthritis. GS should not be expected to have anti-inflammatory effect as DF in the treatment of OA-related effusion.

## Authors Contributions

MK & FK: Designed the Protocol, Performed study procedures and prepared the final manuscript.

YE: Critically reviewed the manuscript for final publication. 

MK: Contributed in manuscript writing.

MK, FK, EB: Involved in clinical management of patients,

IG, HK: Sonologist was involved in clinical Assessment during procedures.

SK: Statistical analysis and review of manuscript.
